# Impact of communicative and critical health literacy on understanding of diabetes care and self-efficacy in diabetes management: a cross-sectional study of primary care in Japan

**DOI:** 10.1186/1471-2296-14-40

**Published:** 2013-03-23

**Authors:** Machiko Inoue, Miyako Takahashi, Ichiro Kai

**Affiliations:** 1 Department of Community Medicine, Teikyo University School of Medicine, 2-11-1, Kaga, Itabashi, Tokyo, 173-8605, Japan; 2 Department of Public Health, Dokkyo Medical University, Tochigi, Japan; 3 School of Public Health, the University of Tokyo, Tokyo, Japan

**Keywords:** Diabetes mellitus, Health literacy, Patient–physician communication, Primary care

## Abstract

**Background:**

The role of a patient’s functional health literacy (HL) has received much attention in the context of diabetes education, but researchers have not fully investigated the roles of communicative and critical HL, especially in primary care. Communicative HL is the skill to extract health information and derive meaning from different forms of communication, and to apply this information to changing circumstances. Critical HL allows the patient to critically analyze information and to use this information to achieve greater control over life events and situations. We examined how HL, particularly communicative and critical HL, is related to the patient’s understanding of diabetes care and self-efficacy for diabetes management in primary care settings. We also examined the impact of patient–physician communication factors on these outcomes, taking HL into account.

**Methods:**

We conducted a cross-sectional observational study of 326 patients with type 2 diabetes who were seen at 17 primary care clinics in Japan. The patients completed a self-administered questionnaire that assessed their HL (functional, communicative, and critical), understanding of diabetes care, and self-efficacy for diabetes management. We also examined the perceived clarity of the physician’s explanation to assess patient–physician communication. Multivariate regression analyses were performed to determine whether HL and patient–physician communication were associated with understanding of diabetes care and self-efficacy.

**Results:**

A total of 269 questionnaires were analyzed. Communicative and critical HL were positively associated with understanding of diabetes care (β = 0.558, 0.451, p < 0.001) and self-efficacy (β = 0.365, 0.369, p < 0.001), respectively. The clarity of physician’s explanation was associated with understanding of diabetes care (β = 0.272, p < 0.001) and self-efficacy (β = 0.255, p < 0.001). In multivariate regression models, HL and perceived clarity of the physician’s explanation were independently associated with understanding of diabetes care and self-efficacy.

**Conclusions:**

Communicative and critical HL and clear patient–physician communication were independently associated with the patient’s understanding of diabetes care and self-efficacy. The potential impact of communicative and critical HL should be considered in communications with, and the education of, patients with diabetes in primary care settings.

## Background

The health literacy (HL) of patients has received much attention as a risk factor for poor adherence to treatment and adverse outcomes in various health care settings [1-5], including diabetes management [2,6-9]. The World Health Organization has defined HL as “the cognitive and social skills which determine the motivation and ability of individuals to gain access to, understand, and use information in ways which promote and maintain good health” [10]. In the context of diabetes care, HL could affect the outcomes of diabetes care through the patient–physician relationship and self-care [11], factors that are closely related to each other [9,12].

Patient–physician communication is associated with adherence to self-management behaviors and with the outcomes of diabetes care [12-15]. Moreover, a trusting patient–physician relationship was reported to have potential beneficial effects on self-efficacy, adherence, and diabetes outcomes [16]. Self-care behaviors, including adherence to diet, exercise, and pharmacotherapy, are crucial for optimal glucose control in patients with diabetes. These self-care behaviors related to the patient’s knowledge and self-efficacy for diabetes self-management [17,18].

As poor HL may limit the patient’s ability to participate in health care [19] and in the exchange of information [20], the association between the HL of patients and diabetes self-management should be explored in detail. Considering the role of the patient–physician relationship in diabetes care, it is important to investigate whether patient–physician communication factors, as well as HL, can affect factors associated with diabetes self-management, such as understanding of diabetes care and self-efficacy for diabetes management [2,21].

To assess HL, it is necessary to consider its dimensions. Nutbeam proposed a three-level model for HL based on the three levels of literacy: functional, communicative/interactive, and critical literacy [22]. According to this model, functional HL consists of the basic skills in reading and writing that are necessary to use health information and health care. Communicative HL is an advanced skill that allows the patient to extract health information and derive meaning from different forms of communication, and to apply the new information to changing circumstances. Critical HL is a more advanced skill that allows the patient to critically analyze information, and to use this information to achieve greater control over life events and situations. Previous studies that examined the impact of HL on diabetes outcomes only assessed functional HL [7,23].

In countries like Japan, where the basic literacy rate is estimated to be 99.8% and 100% of the population completes primary school [24], the roles of higher HL levels, that is, communicative and critical HL, should also be considered. Ishikawa and colleagues developed scales to assess each of the three HL levels among Japanese patients attending a specialty clinic at a university hospital [25]. Their study implied that communicative HL and critical HL, but not functional HL, were associated with self-efficacy for diabetes management in patients seen at a tertiary care center. However, we do not know whether these relationships also exist in primary care settings, when we take into account the characteristics of the patient–physician relationship. Therefore, it is necessary to consider patient–physician communication factors at the same time.

In Japan, the prevalence of diabetes is estimated to be 11.2% (1,067 million) of the total population aged 20–79 years [26], but there were only 4,144 diabetologists in 2010 [27]. Therefore, most patients with diabetes are seen by primary care physicians at the same cost, which is covered by universal health insurance [28]. It is also important to consider that the patient–provider relationship and the communication style in primary care settings could differ from those in tertiary care settings. A long and continuous relationship with a physician could influence communications between the patient and physician, so the relationship could be more trusting and comprehensive compared with the relationship with a diabetologist. As the relationship becomes longer, the number of prescribed drugs, insulin use, and diabetes complications could affect the goal of diabetes education, and the communications could be tailored to these conditions. Some factors, such as social support [29], could be positively associated with the patient’s self-efficacy for diabetes management, while other factors, including the widening use of the Internet [30,31], could affect a patient’s HL and diabetes care. The Internet is frequently used to acquire health-related information in some populations [32-34], and patients may use the Internet to learn about their diseases and how to perform self-care [35]. Theoretically, the use of the Internet for health purposes could be related to each of the three levels of HL; to functional HL, through reading and exchange of information available on the web; to communicative HL, through gathering information and applying the information to self-care; and to critical HL, through critically analyzing the information.

Based on the crucial role of primary care in diabetes self-management and the comprehensive nature of the patient–physician relationship [36], we hypothesized that communicative, critical, and functional HL are strongly associated with a patient’s understanding of diabetes care and self-efficacy for diabetes management in primary care settings, and that patient–physician communication factors may also be associated with these outcomes. Therefore, our aim in this study was to examine these relationships.

## Methods

### Patients

We conducted a cross-sectional observational study using a self-administered questionnaire. First, we recruited participating clinics by mail from 141 family practices and internal medicine clinics (56 health cooperative clinics, 83 Min-Iren clinics, and 2 private clinics) across Japan. The health cooperative clinics and the Min-Iren clinics are supported by the Japanese Health and Welfare Co-operative Federation and the Japan Federation of Democratic Medical Institutions, respectively. There were no differences in the delivery of healthcare or the types of health insurance systems used at these clinics. We chose these organizations as study sites to recruit clinics located in a variety of prefectures (subnational jurisdictions in Japan) to avoid being confined to one locality. To participate in the study, primary care physicians, not diabetologists, had to care for patients with diabetes in the clinic. Among the 19 clinics that agreed to participate in the study, 2 clinics could not complete the research process. As a result, the questionnaires from 17 clinics were considered eligible (13 health cooperative clinics, 3 Min-Iren clinics, and 1 private clinic). The clinics were distributed across 12/47 prefectures.

At each clinic, a physician or a nurse explained the purpose of the study to each consecutive patient with type 2 diabetes who visited the clinic on the survey days. The physicians themselves selected the survey days, taking into account their workload and other factors on the day. Each patient met the following inclusion criteria: age ≥ 20 years and < 75 years old, had been diagnosed with type 2 diabetes ≥1 year ago, was seen regularly at the clinic (at least once every 2 months for the last year, since most patients with diabetes are seen at 4–8 week intervals in Japan [37]), and hemoglobin A_1c_ (HbA_1c_) level ≥ 6.2 % within the last 3 months to confirm that the patient had required medical or lifestyle management for diabetes for at least the last few months. We excluded patients aged ≥ 75 years because the regimens and diabetes education for blood glucose control are usually less intensive for older patients than for younger patients. We also excluded patients who had infrequent contact with their physicians because we aimed to assess the impact of patient–physician communication on diabetes education. Compared with the US and other countries, the consultation time is generally shorter in Japan, as the mean office visit was 6 min long in Japan [38,39]. Considering the shorter consultation time, at least one contact per 2 months for ≥ 1 year was considered the minimum requirement for building a patient–physician relationship in Japan. We also excluded patients with known or apparent cognitive dysfunction, such as those who had previously scored < 23 on the Mini-Mental State Examination (MMSE) [40]. We also excluded those who were unable to answer the questionnaire by themselves because the HL measurement used in this study required the patients to self-assess their ability to use health information.

The eligible patients were provided a questionnaire and were instructed to complete it by themselves at the clinic after the visit or to take it home and complete it later. By responding to the questionnaire, they were considered to have given consent to participate in the study. After completing the questionnaire, the patients mailed it to the data center. The patients were guaranteed the right to withdraw from the study at any time and that their usual care would not be affected by participating in the study. The study was conducted between March and July 2010. To increase the diversity of characteristics of the patients, up to 20 questionnaires were distributed at each clinic. The study protocol was reviewed and approved by the Tokyo Hokuto Health Cooperative Institutional Review Board.

### Health literacy

We measured HL using scales developed in Japan to assess functional, communicative, and critical HL of patients with diabetes. Five, five, and four items were used to assess functional, communicative, and critical HL, respectively [25]. Each item was rated on a 4-point Likert scale, ranging from 1 (never) to 4 (often). In the functional HL subscale, the patients were asked to rate how often they needed someone’s help to read the instructions or leaflets from hospitals/pharmacies, for example. In the communicative HL subscale, the patients were asked to rate, for example, how often they had collected information from various sources since being diagnosed with diabetes. For the critical HL subscale, the patients were asked to rate, for example, how often they had considered the credibility of the information, or had checked whether the information was correct. The scores for functional HL were reversed, such that higher scores indicate higher HL. The scores for the items on each scale were summed and divided by the number of items for that scale to calculate the scale score (theoretical range 1–4). The functional, communicative, and critical HL scales were previously validated in Japanese patients with diabetes [25], and the internal consistency of each scale was adequate (Cronbach’s α = 0.85, 0.81, and 0.69, respectively). Higher scale scores indicate higher HL. There are no cut-off points for classification of adequate/inadequate HL.

### Patient–physician communication

To assess how the patients perceived communication with their physician in terms of information sharing and the decision-making process, we asked the patients to rate the following five items related to communication: “I can ask the physician whatever I would like to know about my disease(s) and physical condition(s),” “the physician knows a lot about my daily life,” “the physician explains other treatment options well,” “the physician’s explanation is clear enough to me,” and “the physician respects my will and ideas at the time of decision-making.” We also assessed overall satisfaction with communication using a sixth item: “I am satisfied with the communication with the physician.” The six items were scored from 1 (I do not agree at all) to 7 (I agree very strongly). These items were not developed or validated as a scale, and the associations among each of six items were very high (*r* = 0.58–0.80, p < 0.001). Therefore, we selected one of the six patient-physician communication variables (“the physician’s explanation is clear enough to me”) as a representative factor in the main analysis because this item was thought to strongly reflect the perceived understandability of the physician’s communication. The other items were not included in the present analyses because they were did not necessarily reflect communication or did not always apply to daily care.

### Understanding of diabetes care and self-efficacy for diabetes management

We examined understanding of diabetes care and self-efficacy for diabetes management to assess the adherence to diabetes self-management. We did not use self-care behaviors because the required self-care behaviors may differ depending on the individual patient’s conditions and the goals of diabetes management. The understanding of diabetes care was assessed using an eight-item scale derived from the Diabetes Quality Improvement Project in a previous study [12]. This scale evaluates the respondent’s perceived understanding of diabetes care and self-management, how to care for feet, and what to do in the event of symptoms of low blood glucose, for example. Each item was scored on a 5-point Likert scale from 1 (I do not know at all) to 5 (I know very well). The scale score was calculated as the mean value of all eight items, with possible scores ranging from 1 to 5. Higher scores imply greater understanding of diabetes care. To use this scale, we first translated it into Japanese and confirmed linguistic validity by back-translation. Reliability and validity were examined in a pilot study of 17 patients. Principal component analysis was conducted, and the proportion of the first principal component was 59.4%, suggesting a single dimension. The test–retest reliability was adequate, with moderate agreement between the test–retest scores (*r* = 0.87, p < 0.001). The internal consistency of the scale was also adequate (Cronbach’s α = 0.89).

Self-efficacy for diabetes management was assessed with a four-item scale of self-care ability in the Diabetes Care Profile, as used in a previous study [25]. The scale evaluates the respondent’s confidence in blood glucose control, weight control, diabetes self-management, and coping with emotions when living with diabetes. Each item was scored from 1 (I do not think I can) to 4 (I strongly think I can). The scale score was calculated as the mean value of all four items, with possible scores ranging from 1 to 4, and higher scores implying greater self-efficacy. The internal consistency was adequate (Cronbach’s α = 0.80).

### Sociodemographic and clinical variables

We obtained demographic data for each patient from the self-reported questionnaire. Clinical data were obtained from separate medical reports provided by the physicians that were matched with the patient questionnaires using an ID number. Each patient reported their height, weight, time since the diagnosis of diabetes, the number of years seen by his/her physician, educational attainment, marital status, occupational status, self-rated economic status, and access to and use of the Internet. We measured the patient’s perceived level of social support using a short-form version of the Multidimensional Scale of Perceived Social Support [41,42]. This is a seven-item scale in which each item is scored from 1 (very strongly disagree) to 7 (very strongly agree). The Japanese version was previously validated [42] and shows adequate internal consistency (Cronbach’s α = 0.89).

As polypharmacy and complex prescription regimens are known to influence treatment adherence [43], and because the status of diabetic complications is associated with HL [25], we also obtained the following clinical information. The physicians reported the age, sex, number of prescribed glucose-lowering drugs, whether or not the patient was on insulin, the most recently measured HbA_1c_, the status of diabetic complications, and whether the complications were managed with or without anti-hypertensive drugs and/or anti-lipid drugs. Diabetic complications included retinopathy, nephropathy, neuropathy, cardiovascular disease, and stroke. These reports were prepared after the patients’ visits. The clinical data for the patients who did not complete the questionnaire were not used in this study.

### Statistical analysis

Pearson’s correlation coefficient was first calculated to determine the relationship between HL and patient–physician communication. We next conducted bivariate analyses to examine the associations between patient–physician communication and the patient’s understanding of diabetes care and self-efficacy. Then, hierarchical linear regression analyses were performed to determine the associations between the three HL scales with the understanding of diabetes care and self-efficacy as outcome variables. Model 1 consisted of bivariate analyses. In model 2, we added sociodemographic and clinical variables that were correlated with the HL scales, the clarity of the physician’s explanation, or with the outcome variables, as possible confounders. Finally, in model 3, we added the clarity of the physician’s explanation to model 2.

To identify the sociodemographic and clinical variables to be included as covariates, we used bivariate analyses to determine their correlations with HL, the clarity of the physician’s explanation, or outcome variables. Age, educational attainment, self-rated economic status, Internet use, social support, time since the diagnosis of diabetes, insulin use, and whether or not they had diabetic complications were correlated with at least one of the three subscales of HL. Internet use and social support were also correlated with the clarity of the physician’s explanation. The number of oral glucose-lowering drugs was weakly correlated with the patient’s understanding of diabetes care (p = 0.08). We included all the variables mentioned above, and sex, as covariates in models 2 and 3.

The variables were entered as follows: age (continuous), sex (male or female), educational attainment (middle school, high school, vocational school/2-year college, or university or higher), self-rated economic status (lower, middle, or upper), Internet use (yes or no), social support (continuous), number of years since the diagnosis of diabetes (continuous), number of oral glucose-lowering drugs (one, two, or three or more), insulin use (yes or no), and diabetic complications (none or any). There was no evidence of multicollinearity among any of the included variables. All statistical analyses were performed using PASW version 18.0. Statistical significance was set at p < 0.05.

## Results

### Descriptive results

Of the 326 questionnaires distributed at 17 clinics, 279 (85.6%) were returned. After checking the demographic data, the responses from 10 patients (3.6%) were excluded from the analysis because these patients did not meet the eligibility criteria (higher age and shorter time since diagnosis). Therefore, 269 questionnaires were analyzed (Table 1). About 20% of patients completed more than 12 years of education. More than 80% of the patients rated their economic status as middle or lower. The Internet was used by 21.9%, and when we included its use by family members, only 49.0% had access to the Internet. These results indicate that half of the participants did not have access to the Internet, even with help from family members, when they wanted to collect information regarding diabetes care. This rate was much lower than the nationally reported rate of 78.2% of the population having access to the Internet at the end of 2010 [30].

**Table 1:** Patient characteristics (N = 269)

Age, mean ± SD (range), years	64.4 ± 7.4 (31–75)
Male gender, n (%)	148 (55.0)
Education, n (%)	
Middle school	83 (30.9)
High school	123 (45.7)
Vocational school/2-year college	23 (8.6)
University or higher	33 (12.3)
No answer	7 (2.6)
Self-rated economic status, n (%)	
Lower	85 (31.6)
Middle	142 (52.8)
Upper	38 (14.1)
No answer	4 (1.5)
Internet use by themselves, n (%)	
Yes	58 (21.6)
No	205 (76.2)
No answer	6 (2.2)
Social support, mean ± SD (range)	5.1 ± 1.3 (1–7)
Time since the diagnosis of diabetes, mean ± SD (range), years	11.0 ± 9.4 (1–48)
Hemoglobin A_1c_, mean ± SD (range), %	7.6 ± 1.1 (5.6–13.1)
BMI, mean ± SD (range), kg/m^2^	24.8 ± 3.9 (15.6–43.6)
Understanding of diabetes care, mean ± SD (range)	3.78 ± 0.75 (1–5)
Self-efficacy for diabetes management, mean ± SD (range)	2.69 ± 0.58 (1–4)
Number of oral glucose-lowering drugs, n (%)	
None	77 (28.6)
One	107 (39.8)
Two	62 (23.0)
More than three	23 (8.6)
Insulin use, n (%)	59 (21.9)
Complications, n (%)	
None	153 (56.9)
Retinopathy	45 (16.7)
Nephropathy	75 (27.9)
Neuropathy	20 (7.4)
Ischemic heart disease	19 (7.1)
Stroke	15 (5.6)

The mean scores for each HL subscale are shown in Table 2. Among the three subscales, functional HL had the highest mean score, and critical HL had the lowest mean score. Among the HL scales, communicative HL and critical HL were highly correlated (*r* = 0.752, p < 0.001). The overall score for patient–physician communication, as determined by the “clarity of physician’s explanation,” was high, as the mode and the median values were both 6 out of 7 points. Patient–physician communication was correlated with communicative HL (Table 2). The clarity of the physician’s explanation was also positively associated with understanding of diabetes care and self-efficacy in bivariate analyses (β = 0.272 and 0.255, respectively, p < 0.001) (data not shown). There were no significant differences in HL scores or patient–physician communication items among the participating clinics.

**Table 2:** Correlations among health literacy and patient–physician communication

		**Correlation coefficients**			
	**Mean ± SD**	**Functional HL**	**Communicative HL**	**Critical HL**	**Clarity of the physician’s explanation**
HL					
Functional HL	3.36 ± 0.58	1	0.074	−0.048	0.035
Communicative HL	2.65 ± 0.68		1	0.752†	0.231†
Critical HL	2.28 ± 0.59			1	0.055
Clarity of the physician’s explanation	5.72 ± 1.04				1

Pearson’s correlation coefficients are shown. *p < 0.05; †p < 0.01. The score range was 1–4 for HL and 1–7 for the clarity of the physician’s explanation.

HL: health literacy.

In bivariate analyses, Internet use was associated with higher functional HL (p < 0.001) and with communicative HL (p = 0.004), but not with critical HL (p = 0.112). Internet use was not associated with understanding of diabetes care (p = 0.201) or self-efficacy for diabetes management (p = 0.947). Social support was associated with communicative HL (p = 0.002), critical HL (p = 0.002), and the clarity of the physician’s explanation (p = 0.005), but not with functional HL (p = 0.900). Social support was also significantly associated with self-efficacy for diabetes management (p < 0.001). Insulin use was significantly associated with higher communicative HL (p = 0.002), critical HL (p = 0.017), and understanding of diabetes care (p < 0.001).

### Association of HL and patient–physician communication with understanding of diabetes care and self-efficacy for diabetes management

Tables 3 and 4 show the multivariate linear regression models with understanding of diabetes care and self-efficacy for diabetes management as the outcome variables, respectively. In Table 3, functional, communicative, and critical HL were positively associated with understanding of diabetes care in the unadjusted (model 1) and adjusted (model 2) models. Including the clarity of the physician’s communication (i.e. model 3) did not significantly change the β coefficients for HL. The clarity of the physician’s communication was significant in model 3, and the β coefficients did not change significantly compared with the model without HL. Among the covariates, insulin use was consistently associated with higher understanding of diabetes care (p < 0.001) in models including any of the subscales of HL. In model 3, the number of oral glucose-lowering drugs was positively and significantly associated with understanding of diabetes care (p = 0.037) in the model including critical HL, but not in the models including functional or communicative HL. The other covariates were not significant in any models.

**Table 3:** Linear regression models with understanding of diabetes care as the outcome variable

	**HL**					
	**Functional HL**		**Communicative HL**		**Critical HL**	
	**β**	**p**	**β**	**p**	**β**	**p**
Model 1	0.155	0.015	0.558	< 0.001	0.451	< 0.001
Model 2	0.176	0.011	0.503	< 0.001	0.429	< 0.001
Model 3	0.172	0.011	0.479	< 0.001	0.426	< 0.001

Model 1: unadjusted.

Model 2: adjusted for age, sex, education, economic status, Internet use, social support, time since the diagnosis of diabetes, number of oral glucose-lowering drugs, insulin use, and diabetic complications.

Model 3: model 2 plus the clarity of the physician’s explanation.

HL: health literacy.

**Table 4:** Linear regression models with self-efficacy for diabetes management as the outcome variable

	**HL**					
	**Functional HL**		**Communicative HL**		**Critical HL**	
	**β**	**p**	**β**	**p**	**β**	**p**
Model 1	0.004	0.948	0.365	< 0.001	0.369	< 0.001
Model 2	0.015	0.833	0.312	< 0.001	0.300	< 0.001
Model 3	0.010	0.891	0.292	< 0.001	0.298	< 0.001

Model 1: unadjusted.

Model 2: adjusted for age, sex, education, economic status, internet use, social support, time since the diagnosis of diabetes, number of oral glucose-lowering drugs, insulin use, and diabetic complications.

Model 3: model 2 plus the clarity of the physician’s explanation.

HL: health literacy.

In Table 4, communicative and critical HL were positively associated with self-efficacy for diabetes management in the unadjusted (model 1) and adjusted (model 2) models. Similar to Table 3, including the clarity of the physician’s communication (i.e. model 3) did not significantly change the β coefficients for HL. The clarity of the physician’s communication was also significant in model 3, and the β coefficients did not change significantly compared with the model without HL. Social support (p = 0.002), time since the diagnosis of diabetes (p = 0.028), and the absence of diabetic complications (p = 0.046) were significantly associated with higher self-efficacy in the model that included functional HL. In the model that included critical HL, social support was significantly associated with self-efficacy (p = 0.028).

The results of the analyses using other patient–physician communication items, instead of the clarity of the physician’s explanation, did not differ substantially from the results reported in the Tables 2, 3, and 4.

## Discussion

This study is the first to measure the three dimensions of HL and patient–physician communication in patients with diabetes in primary care settings. We found that functional HL was weakly associated with understanding of diabetes care; however, it was not associated with self-efficacy for diabetes management. On the other hand, communicative and critical HL were both significantly associated with the patient’s understanding of diabetes care and self-efficacy. These findings were similar to those of a previous study conducted at a university hospital in Japan [25,44], despite the differences in practice settings and social demographics of the study participants. Greater proportions of participants in our study in primary care settings reported having lower educational attainment and lower self-rated economic status than the participants in the previous study. In our study, we also considered the role of patient–physician communication and other covariates in the regression models, which had not been conducted before. Our results support the concept that functional HL plays a weaker role than communicative and critical HL in the relationship between HL and diabetes management in a population with relatively high functional HL, as in our participants in Japan. While functional HL is thought to affect the way patients use the information necessary for diabetes self-management [45-47], our findings clearly demonstrate the potential roles of communicative and critical HL in diabetes care.

We also found that in primary care, patient–physician communication was associated with both diabetes understanding and self-efficacy for diabetes management. Although patient–physician communication and communicative HL were correlated with each other, they were independently associated with the outcome variables when they were included in the same model. As patient empowerment has become a new paradigm in the patient–physician relationship in the context of diabetes care [48], family physicians should be encouraged to reinforce a relationship with patients based on clear communication to encourage better diabetes management.

Our results also revealed interesting associations among social support, HL, and self-efficacy. Social support was associated with self-efficacy in bivariate analyses, although the association disappeared when communicative HL was included in the model. The role of social support in diabetes management in the context of HL should be examined in further studies.

Internet use was not significantly associated with the outcome variables in this study. The surprisingly low prevalence of Internet use among our patients might explain these results. According to a population-based study by Takahashi and colleagues [34], to acquire health-related information, younger people, people with higher education levels, and people with higher household incomes were more likely to access the Internet. Considering our patients’ relatively high age and low educational level, it is understandable that the Internet was not easily or frequently accessed as a source of health-related information.

Our study has several limitations. First, it is possible that patients with very low HL declined to participate in this study. Meanwhile, patients with a favorable relationship with their physicians may have been selected because physicians were involved in identifying patients to participate in this study, although physicians were instructed to approach consecutive patients. The patients’ assessments of their physicians’ communications were very high, and the patients may have preferred giving socially desirable responses. These factors should be considered when interpreting the results. Second, HL and understanding of diabetes care were evaluated by self-assessed scales rather than objective methods. Additionally, most of the patients completed the questionnaire at home. Although they were asked to answer the questionnaire by themselves, it is possible that some of the patients received some assistance. Objective methods to measure HL and knowledge of diabetes care should be used in future studies. In addition, we used the item “the clarity of the physician’s explanation” to assess patient–physician communication. Therefore, more comprehensive measures should be used to examine patient–physician communication in long-term relationships in primary care. Finally, there are some international differences in the way diabetes care is provided. The duration of office visits is generally very short in Japan, but patients see their physicians more frequently in Japan than in the US healthcare system, for example [37-39]. Therefore, it will be important to investigate whether our findings are generalizable to populations in different healthcare systems and other cultural backgrounds.

## Conclusion

In conclusion, despite the limitations described above, our results are important because we have assessed communicative and critical HL, as well as perceived patient–physician communication. These factors were independently associated with the understanding of diabetes care and with self-efficacy for diabetes management. Primary care physicians and other healthcare professionals should endeavor to use clear communication when providing diabetes education, and acknowledge the potential impact of communicative and critical HL on diabetes management, even for patients whose functional HL seems to be adequate.

## Competing interests

The authors declare that they have no competing interests.

## Authors’ contributions

MI conceived the study, collected the data, performed the statistical analysis, and drafted the manuscript. MT and IK contributed to the study design, data interpretation and review of the manuscript. All authors read and approved the final manuscript.

## Pre-publication history

The pre-publication history for this paper can be accessed here:

http://www.biomedcentral.com/1471-2296/14/40/prepub
